# CapG promoted nasopharyngeal carcinoma cell motility involving Rho motility pathway independent of ROCK

**DOI:** 10.1186/s12957-022-02808-7

**Published:** 2022-10-19

**Authors:** Ying Fu, Xiuzhi Zhang, Xujun Liang, Yongheng Chen, Zhuchu Chen, Zhefeng Xiao

**Affiliations:** 1 Department of Pathology, NHC Key Laboratory of Cancer Proteomics, National Clinical Research Center for Geriatric Disorders, Xiangya Hospital, Central South University, Changsha, 410008 Hunan China; 2 Department of Pathology, Henan Medical College, Zhengzhou, 451191 Henan China

**Keywords:** CapG, Nasopharyngeal carcinoma, Motility, ROCK, Rac1

## Abstract

**Background:**

Gelsolin-like capping actin protein (CapG) modulates actin dynamics and actin-based motility with a debatable role in tumorigenic progression. The motility-associated functions and potential molecular mechanisms of CapG in nasopharyngeal carcinoma (NPC) remain unclear.

**Methods:**

CapG expression was detected by immunohistochemistry in a cohort of NPC tissue specimens and by Western blotting assay in a variety of NPC cell lines. Loss of function and gain of function of CapG in scratch wound-healing and transwell assays were performed. Inactivation of Rac1 and ROCK with the specific small molecular inhibitors was applied to evaluate CapG’s role in NPC cell motility. GTP-bound Rac1 and phosphorylated-myosin light chain 2 (p-MLC2) were measured in the ectopic CapG overexpressing cells. Finally, CapG-related gene set enrichment analysis was conducted to figure out the significant CapG-associated pathways in NPC.

**Results:**

CapG disclosed increased level in the poorly differentiated NPC tissues and highly metastatic cells. Knockdown of CapG reduced NPC cell migration and invasion in vitro, while ectopic CapG overexpression showed the opposite effect. Ectopic overexpression of CapG compensated for the cell motility loss caused by simultaneous inactivation of ROCK and Rac1 or inactivation of ROCK alone. GTP-bound Rac1 weakened, and p-MLC2 increased in the CapG overexpressing cells. Bioinformatics analysis validated a positive correlation of CapG with Rho motility signaling, while Rac1 motility pathway showed no significant relationship.

**Conclusions:**

The present findings highlight the contribution of CapG to NPC cell motility independent of ROCK and Rac1. CapG promotes NPC cell motility at least partly through MLC2 phosphorylation and contradicts with Rac1 activation.

**Supplementary Information:**

The online version contains supplementary material available at 10.1186/s12957-022-02808-7.

## Background

Nasopharyngeal carcinoma (NPC) is a highly invasive and metastatic cancer arising from the nasopharynx epithelium, which is particularly prevalent in southern China and Southeast Asia [1]. A combination of ethnic, genetic, EBV infection, and environmental factors might affect nasopharyngeal carcinoma pathogenesis [1]. One of the unique clinical features of NPC that differs from most other head and neck squamous cell carcinomas is the propensity for early subclinical dissemination and distant metastasis [2]. Despite the excellent local control with modern chemoradiotherapy, locoregional recurrence and distant failure remain a challenging problem [3]. It was estimated that 21.3% of the patients with nonmetastatic NPC developed failure mainly for distant metastasis within 5 years after primary treatment [4]. Thus, further understanding of the molecular rationale of NPC cell motility is needed for the development of better treatment strategies and for the survival improvement.

Gelsolin-like actin-capping protein (CapG, also known as gCAP39 or MCP) is a ubiquitous nuclear-cytoplasmic actin filament capping protein of the gelsolin superfamily [5]. It is expressed at moderately high levels in most cell types (except platelets), particularly abundant in macrophages [6]. Despite the lack of severing activity in contrast to gelsolin, CapG overexpression also increased fibroblast motility [7]. It reversibly binds to and is regulated by calcium ion and polyphosphoinositides, such as phosphatidylinositol 4,5-bisphosphate (PIP2). Increasing intracellular [Ca^2+^] or reduction in local [PIP2] would allow CapG to cap the barbed filament ends, which is a key control step in the regulation of actin polymerization, propulsive force generation, and actin-based motility [8]. Capping of filaments would probably serve to prevent misdirected actin filament growth, thereby allowing the more efficient production of directional force and avoiding chaos [9].

CapG has been reported of oncogenic functions in a variety of cancers [10–12]. Increased CapG was found in human oral premalignant lesions [13] and in the interface zone of breast cancer, the region between the invading tumor front and normal tissue [14], indicating CapG involvement in early carcinogenesis and margin invasion. Nevertheless in a few studies, CapG was found as a tumor suppressor gene [15, 16], which suggested a controversial role.

Rho-family small GTPases are key regulators of cytoskeletal dynamics, most of which switch between an active GTP-bound form and an inactive GDP-bound form. For instance, the activation of Rho and Rac leads to the assembly of contractile actin-myosin filaments and protrusive actin-rich lamellipodia, respectively [17]. Actin-myosin contractile force promotes an amoeboid form of movement, while the protrusive force of actin polymerization drives an elongated mode of movement [18]. Rho and Rac act mutually antagonistically on a number of effectors such as protein kinases and actin-binding proteins to regulate the conversion between these two modes of cell movement [19]. For instance, rho-associated protein kinases (ROCK) I and II were the effectors of Rho to mediate actin-myosin contractility through their effects on the phosphorylation of myosin light chain (MLC), resulting in the repression of Rac activity via ARHGAP22 to suppress elongated motility [19, 20].

Motility-related function and potential mechanisms of CapG in NPC have remained unclear. Our former study found CapG level to be higher in the NPC histopathological subtype of non-keratinizing squamous cell carcinoma (NKSCC) than in the keratinizing squamous cell carcinoma (KSCC) by quantitative proteomics tools [21]. In the present study, we validated CapG expression in the NPC tissue specimens of different histopathological subtypes and seven of NPC cell lines and further explored its motility-related functions and molecular mechanisms involved in the small Rho-GTPase signaling. The study would deepen our understanding of NPC cell motility mechanisms as well as provide novel clues to NPC therapeutic strategies.

## Methods

### Patient tissue specimens

With the approval of the Ethic Committee of Xiangya Hospital of Central South University, a total of 46 formalin-fixed, paraffin-embedded primary NPC specimens and 15 normal nasopharyngeal epithelial tissue samples (NNET) obtained from the Department of Pathology of Xiangya Hospital were used for the immunohistochemistry assay. NPC specimens contained 34 of NKSCC subtypes and 12 of KSCC. All patients were first diagnosed with NPC both histologically and clinically between 2013 and 2016, and none was subjected to radiotherapy or chemotherapy before biopsy sampling.

### Cell culture and transfection

Seven human NPC cell lines of CNE1 (highly differentiated), CNE2 (poorly differentiated), 5-8F (highly metastatic), 6-10B (lowly metastatic), HNE1, HNE2, and HNE3 originate from the cell repository of the Cancer Research Institute of Central South University. All the cell lines were cultured in RPMI 1640 medium (Gibco, Suzhou, China) supplemented with 10% (v/v) fetal bovine serum (Gibco, NY, USA). The cells were maintained in a humidified atmosphere with 5% CO_2_ at 37 °C.

RNA interference or stable overexpression of CapG was conducted to the NPC cell lines. Two pairs of small interfering RNA (siRNA) duplexes targeting CapG (s228278#1: sequence 5′-GAU AUC UGA UGA CUG CUU Utt-3′; s2379#2: sequence 5′-GAG UCA GCA UUU CAC AAG Att-3′) and negative control (NC) siRNA were designed and synthesized by Ambion (Carlsbad, USA). HNE3 and 5-8F cells were disseminated into 6-well plates at a concentration of 0.5 × 10^6^ cells per well containing 2 ml serum-free medium for 24 h prior to transfection. Cell transfection was achieved using Lipofectamine RNAiMAX reagent (Invitrogen, CA, USA) according to the manufacturer’s instruction. Briefly, 35 nM siRNA duplex with RNAiMAX reagent was incubated with cells for 6 h at 37 °C, 5% CO2, and afterwards, the medium was replaced with fresh culture medium. Cells were harvested at 48 h post transfection, and Western blot assay was taken to determine the transfection efficiency. For CapG stable overexpression, the full-length sequence of CapG (NM_001747) was cloned into the empty pLent-EF1a-FH-CMV-GP vector (ViGene Biosciences, Beijing, China). HNE1 and 6-10B cells were infected with recombinant lentivirus-transducing units or empty vectors plus 10 μg/ml ADV-HR reagent (ViGene Biosciences, Beijing, China) according to the manufacturer’s instruction. Stably transfected HNE1 cells and 6-10B cells were selected by 3.5 μg/ml and 8 μg/ml puromycin (ab141841, Abcam, Cambridge, UK) respectively for 3 weeks. Ectopic CapG overexpression was validated by Western blot.

### Immunohistochemistry staining and Western blotting assay

Immunohistochemistry study was performed using a standard streptavidin-biotin-peroxidase complex method described previously [21]. Four micrometer-thick tissue sections were deparaffinized, treated with antigen retrieval solution (10 mmol/l sodium citrate buffer; pH 6.0), and incubated with rabbit anti-CapG polyclonal antibody (1:200; ab155688, Abcam, Cambridge, UK) overnight at 4 °C, followed by secondary antibody incubation and color development. In negative controls, the primary antibody was omitted. CapG staining was blindly scored according to the percentage of positive staining in the whole section for each case (0 = no positive staining; 1 = 1–30% positive; 2 = 31–60% positive; 3 = 61–100% positive) and the intensity (0 = negative, 1 = mild staining, 2 = moderate staining, 3 = intense staining), as described previously [21]. Finally, the immunoreactivity score (ranging from 0 to 6) was obtained by adding the area score and the intensity score for each case.

For Western blot assay, cells were harvested and lysed in cold RIPA buffer (150 mM NaCI, 0.1% SDS, 1% Triton X-100, 1% sodium deoxycholate, 1 mM EDTA, 50 mM Tris, pH 7.4) containing 1 mM PMSF and phosphatase inhibitor cocktail (CW2383s, CWbiotech, Beijing, China). Protein supernatant was collected after centrifugation at 4 °C, 12,000 r.c.f. for 20 min; the concentration was determined using a BCA Protein Assay kit (Beyotime Biotechnology, Shanghai, China), and the protein in equal quantity was denatured at 100 °C for 10 min with SDS-PAGE Sample Loading Buffer (Beyotime Biotechnology, Shanghai, China). Denatured protein was separated via 10% SDS polyacrylamide gel electrophoresis and transferred to PVDF membrane (0.22 μm, Merck Millipore, MA, USA). Membrane was blocked with TBST solution (20 mM Tris, 137 mM NaCl, 0.1% Triton X-100, pH 7.6 ± 0.1) containing 5% nonfat milk for 1 h at room temperature, followed by incubation with specific primary antibodies at 4 °C for 16 h. The primary antibodies included alpha tubulin (1:2000; ab52866, Abcam, Cambridge, UK), CapG (1:1000; ab155688, Abcam, Cambridge, UK), MLC2 (1:2000; 10906-1-AP, Proteintech, IL, USA), and phospho-MLC2 (p-MLC2) (1:1000; no. 3674, Cell Signaling Technology, MA, USA). Immunoblotting of alpha tubulin served as loading control. After the unbound primary antibodies were washed away, proper secondary antibodies labeled with horseradish peroxidase (HRP) were incubated for 1 h at room temperature. 3,3N-Diaminobenzidine tetrahydrochloride (DAB) was applied to staining, and an electrochemiluminescence system (Tanon, Shanghai, China) was used for chemiluminescence detection.

### Scratch wound healing assay and transwell migration/invasion assay

Before application of the small molecular inhibitors, the loss and gain function of CapG in NPC cell migration and invasion were assessed using scratch wound healing assay and transwell migration/invasion assay. For scratch wound healing assay, cells were cultured to confluent monolayer in 6-well plates, three parallel scratches were drawn per well with a 200 μl sterile pipette tip, and culture medium was replaced with serum-free RPMI-1640. Spread of wound closure was observed by taking the images at various times after wounding for the same eight spots, localized on the underside of the well by a marker. Cell migration rate was calculated according to the equation listed below: cell migration rate (%) = (1—the distance after healing/the distance before healing) ×100. It was repeated twice; thus, a total of 16 views of each group entered statistical analysis.

For transwell migration/invasion assay, cells were suspended in serum-free RPMI-1640 medium of 200 μl at a concentration of 5 × 10^4^ for the transfected 5-8F/6-10B cells, 2.5 × 10^4^ for transfected HNE1/HNE3 cells, and the corresponding NC groups respectively, and the inhibitors pretreated groups were also tested. Cells were seeded into the Matrigel-coated chambers (8 μm, Corning, NY, USA) or non-coated chambers. The bottom chamber was complemented with the culture medium containing 10% fetal bovine serum. After 48 h of incubation at 37 °C with 5% CO2, the non-invading cells or non-migrating cells on the upper surface of the chamber filter membrane were scrubbed and removed with cotton swabs, while the remaining cells that migrated or invaded to the lower surface of the filter membrane were fixed in 100% methanol and stained with crystal violet (0.5%, Sigma-Aldrich, MO, USA). Fixed cells were counted and photographed under an inverted microscope. The experiment was repeated twice for each group, and four visual fields per chamber were statistically analyzed.

### Application of the small molecular inhibitors

Two small molecular inhibitors of NSC23766 (Selleck, TX, USA) and Y-27632 (Selleck, TX, USA) were adopted. NSC23766 is a specific inhibitor of a subset of guanine nucleotide exchange factor-mediated activation of Rac1, without interfering with Cdc42 or RhoA activity [22]. Y-27632 is a ROCK inhibitor targeting both of the ROCK isoforms of ROCK1 and ROCK2 [23]. Ectopic CapG overexpressing 6–10B and HNE1 cells and the matched NC groups were seeded into 6-well plates and grew to approximately 80% confluence in complete culture medium. Then Y-27632 was added into CapG overexpressing 6-10B cells and the NC group at 50 μM. A mixed cocktail of 50 μM of Y-27632 and 100 μM of NSC23766 was added into CapG overexpressing 6-10B cells and the NC group. For HNE1 cells, it was 25 μM of Y-27632 into CapG overexpressing HNE1 cells and the NC group and a combination of 25 μM of Y-27632 and 50 μM of NSC23766 into CapG overexpressing HNE1 cells and the NC group. After 24-h incubation, the culture medium was replaced with serum-free RPMI-1640 medium, followed by transwell migration/invasion assays.

### Rac1 activity assay

Rac1 activity (GTP Rac1/total Rac1) was determined by a pull-down assay using a Rac1 activation assay kit (STA-401-1, Cell Biolabs, CA, USA) according to the manufacturer’s protocol. Assay takes the advantage of the p21-binding domain of human p21-activated kinase 1 as a probe, which specifically binds to the GTP-bound form of Rac and shows negligible binding to the GDP-bound form [24]. Subsequently, the precipitated GTP-Rac1 is detected by Western blotting analysis using an anti-Rac1 antibody. Briefly, when ectopic CapG overexpressing 6-10B and HNE1 cells and the NC groups grew to approximately 90% confluence in 10-cm plates, they were serum starved for 24 h. Afterwards, the cells were lysed, quantified, and incubated with the agarose beads probe. Binding was allowed to proceed for 1.5 h at 4 °C with gentle agitation. After incubation, the beads were centrifuged and washed three times to remove unbound material. GTP-Rac1 was detached from the beads by boiling the samples in 2× SDS-PAGE sample buffer for 5 min and subjected to immunoblotting using an anti-Rac1-specific monoclonal antibody. Total Rac1 protein in the pre-immunoprecipitated cell lysate was also visualized simultaneously.

### Gene set enrichment analysis (GSEA)

Gene expression profiling datasets of NPC in the Gene Expression Omnibus database (GEO) were evaluated and selected GSE102349 for the study as it contains a maximum number of 113 NPC samples with a procurement standard of a tumor cell content of at least 50% [25]. Correlations of CapG with all the other genes in GSE102349 were investigated in R studio (version 1.3.1093) by Spearman correlation analyses. To characterize the guilt of CapG association, GSEA was performed over the whole genes ranked by their Spearman’s correlation coefficients with CapG. Annotated gene set “BioCarta_2016” from the gene set library of Enrichr [26] was selected as a reference, which contained 237 molecular pathways including Rho cell motility signaling pathway and Rac1 cell motility signaling pathway. The GSEA tool was from the clusterProfiler package [27]. *p*-values of < 0.05 and false discovery rate of *q* < 0.05 were regarded as statistically significant.

### Statistical analysis

Kruskal-Wallis test was used for the multiple comparisons of CapG expression in the tissue samples. Relative levels of the proteins in the cell lines were expressed as mean ± SD of three independent experiments. One-way analysis of variance test was used for the comparisons between different groups. For all the analyses, *p* < 0.05 was considered as statistically significant.

## Results

### CapG shows higher expression in the more malignant NPC cell lines and histopathological subtypes

**Fig. 1:**
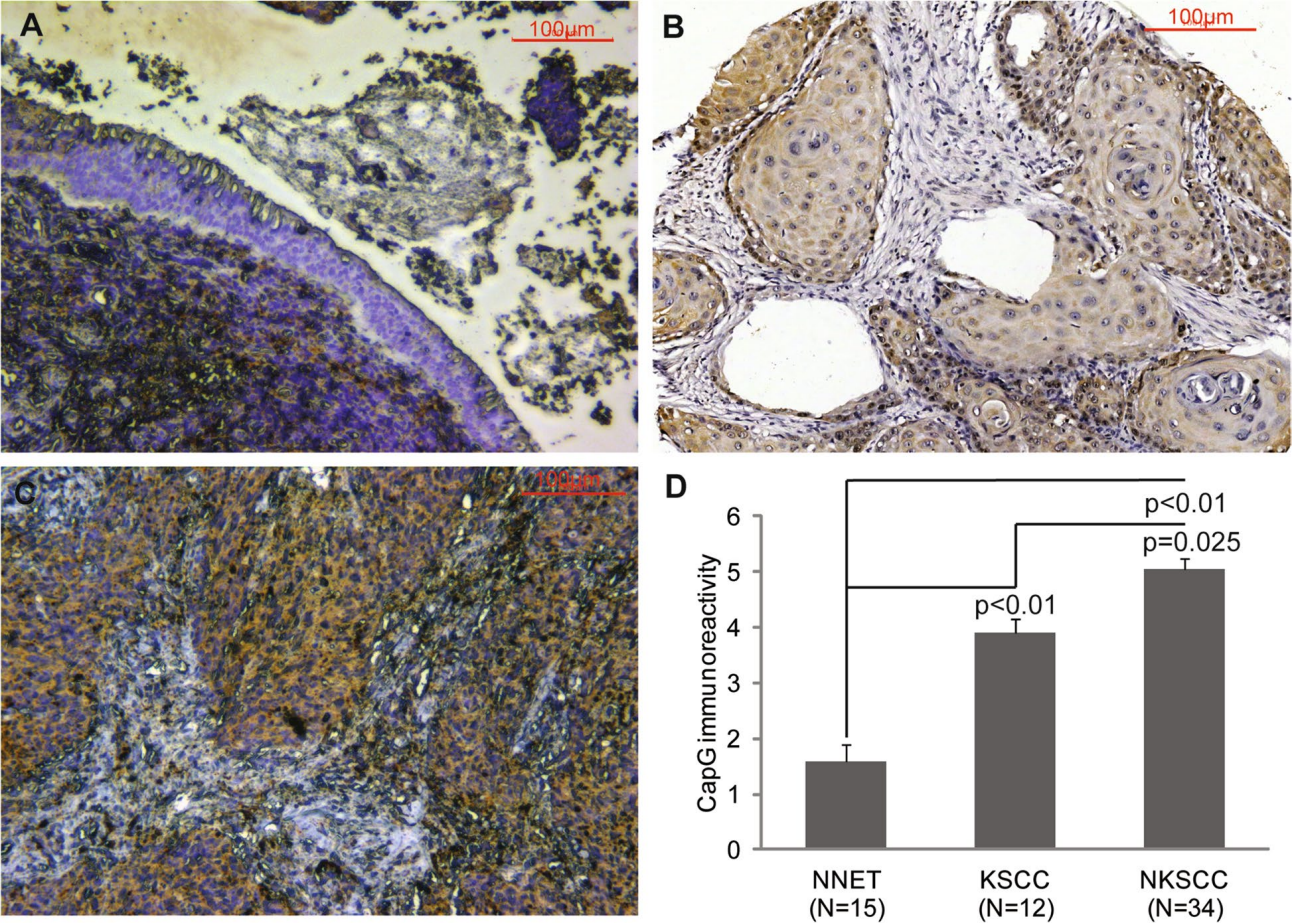
Expression of CapG in a Chinese NPC cohort. **A**-**C** Immunohistochemical staining patterns of CapG with 100 μm scale bars in NNET (**A**), KSCC (**B**), and NKSCC (**C**). **D** The immunoreactivity scores among NNET, KSCC, and NKSCC are represented as mean ± SD. NNET, normal nasopharyngeal epithelial tissue; KSCC, keratinizing squamous cell carcinoma; NKSCC, non-keratinizing squamous cell carcinoma

CapG expression was determined by immunohistochemistry in different differentiated NPC specimens including 12 cases of KSCC and 34 cases of NKSCC, coupled with 15 cases of NNET. It was found that CapG was predominantly expressed in the malignant cells rather than in the normal nasopharyngeal epithelium (Fig. 1). In different histopathological subtypes, CapG was higher in the cytoplasm and nuclei of the NKSCC than in that of the KSCC (Fig. 1).

**Fig. 2:**
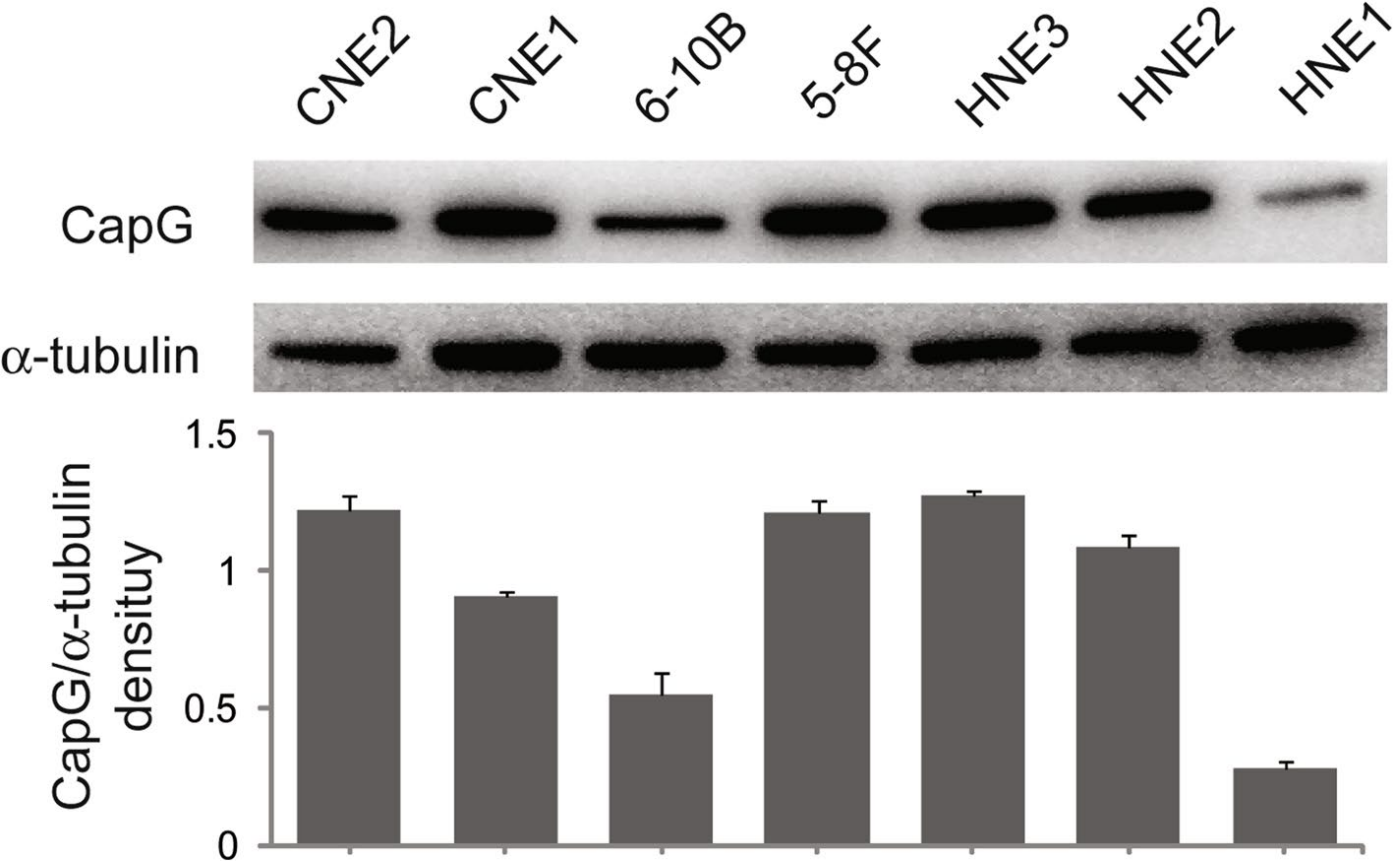
CapG expression in a panel of NPC cell lines. α-Tubulin was used as a loading control. Grouped blots were cropped from different parts of the same gel and from different exposures

In the seven NPC cell lines, immunoblotting assay revealed higher levels of endogenous CapG in CNE2, 5-8F, HNE2, and HNE3 than in CNE1, 6-10B, and HNE1 (Fig. 2). CapG increased in the high-grade malignant cell lines such as highly metastatic 5-8F and poorly differentiated CNE2. Cell lines with the biggest disparity in CapG level were selected for next experiments. 5-8F and HNE3 cells were selected for CapG knockdown; 6–10B and HNE1 cells were used for ectopic CapG overexpression.

### CapG accelerates cellular migration and invasion in vitro

**Fig. 3:**
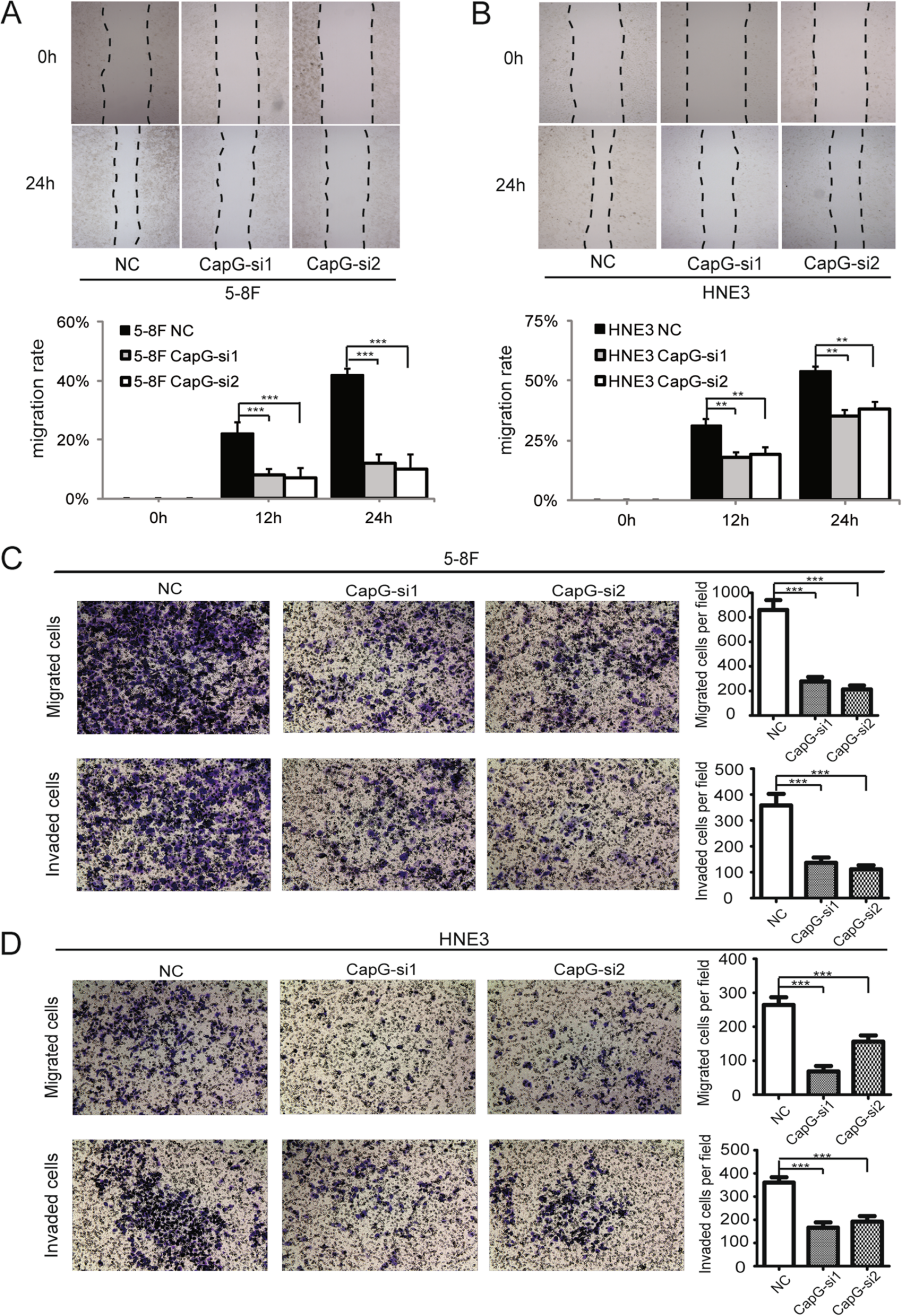
CapG knockdown attenuated the aggressive phenotypes of the NPC cells in vitro. **A** and **B** Wound-healing assay showed the migration positions of 5–8 F and HNE3 cells with CapG-siRNA knockdown and the NC groups at 0 and 24 h after wounding. Bar chart showed the proportion of the migration size at 0, 12, and 24 h. **C** and **D** Transwell assays showed the migratory or invasive cells of 5–8 F and HNE3 cells with CapG-siRNA knockdown and the NC groups. Representative fields were photographed. NC, negative control. **p < 0.01, ***p < 0.001

Loss and gain of function of CapG in NPC cell migration and invasion were examined in vitro. CapG was transiently knocked down in 5-8F and HNE3 cells using two pairs of small interfering RNA of si-228278#1 and si-2379#2. Knockdown efficiency was measured by Western blotting. CapG protein levels were substantially reduced by more than 65% in the four CapG-siRNA transfected cell lines than in the NC groups (Additional file 1: Fig. 1A). Wound-healing assay demonstrated that knockdown of CapG caused an apparent suppression of migration at the edge of exposed regions in both 5-8F and HNE3 cells (Fig. 3 A and B). Both the CapG knockdown cell lines exhibited a dramatic decrease relative to the NC groups (*p* < 0.001, Fig. 3 C and D) in the number of cells that migrated through the non-coated chamber membrane or invaded through a thin layer of reconstituted extracellular matrix (Matrigel).

**Fig. 4:**
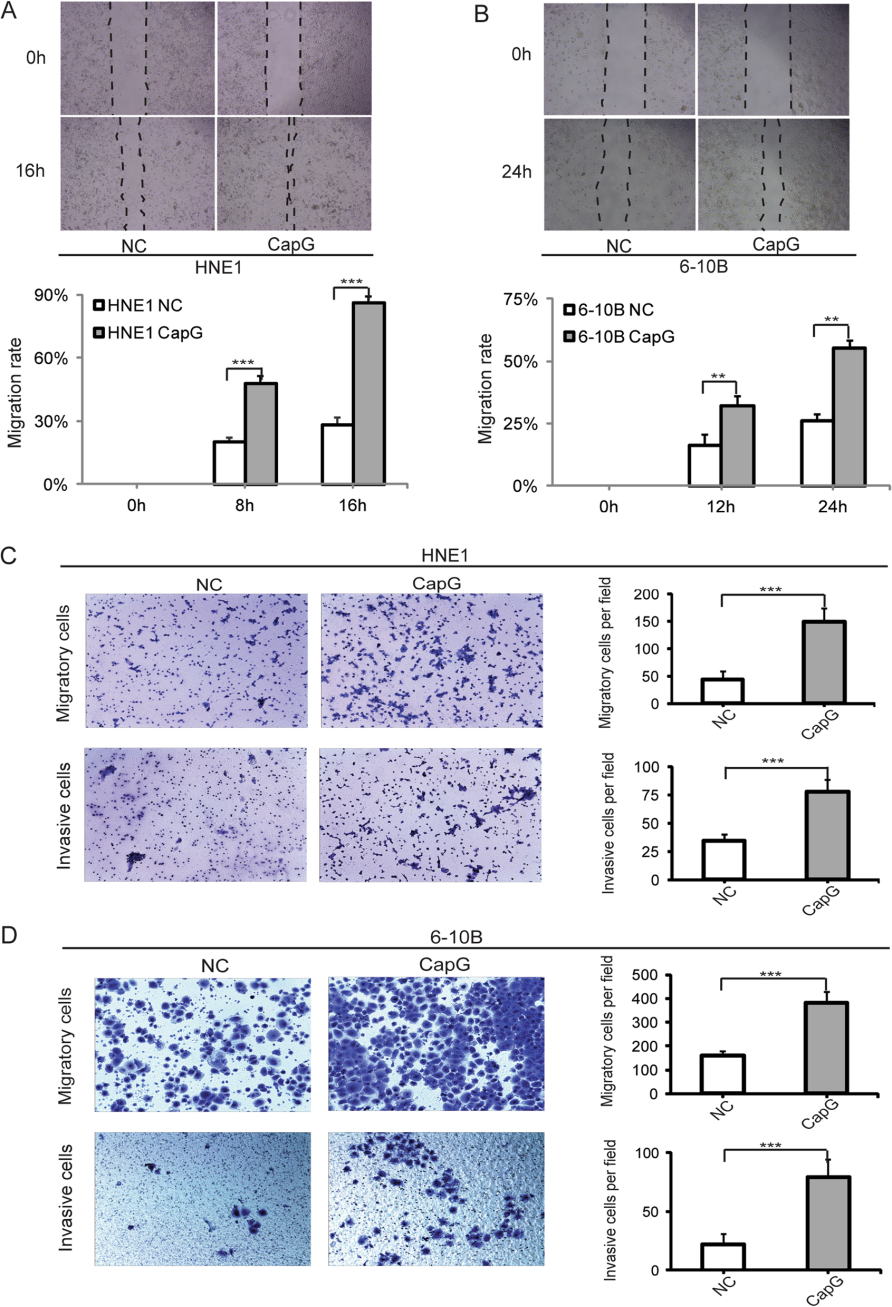
CapG induced the aggressive phenotypes of the NPC cells in vitro. **A** and **B** Wound-healing assay showed the migration positions of HNE1 and 6–10 B cells with ectopic CapG overexpression and the NC groups at 0 and 16 h for HNE1, 0 and 24 h for 6–10 B after wounding. Bar chart showed the proportion of the migration size. **C** and **D** Transwell assay showed the migratory and invasive cells of HNE1 and 6-10B cells. Representative fields were photographed. NC, negative control. **p < 0.01, ***p < 0.001

Cell migration and invasion in HNE1 and 6-10B cells with ectopic overexpression of CapG were evaluated. Western blotting validated CapG expression level in the recombinant vector transfected cells to more than twice as that in the NC groups, as shown in Additional file 1: Fig. 1B. In the wound-healing assay, the rate of wound healing was markedly faster in the CapG-overexpressing HNE1 and 6-10B cells than in the NC cells. At 16-h and 24-h post-scratching, the CapG-overexpressing HNE1 cells and 6-10B cells had migrated approximately 90% and 60% of the open gap measured at 0 h, respectively. The NC cells had migrated about 30% and 25%, respectively (Fig. 4 A and B). Transwell migration and invasion assay showed that CapG-overexpressing HNE1 and 6-10B cells were much more migratory and invasive than the NC groups (Fig. 4 C and D).

### Overexpression of CapG promotes NPC cell migration and invasion independently of ROCK and Rac1 in vitro

**Fig. 5:**
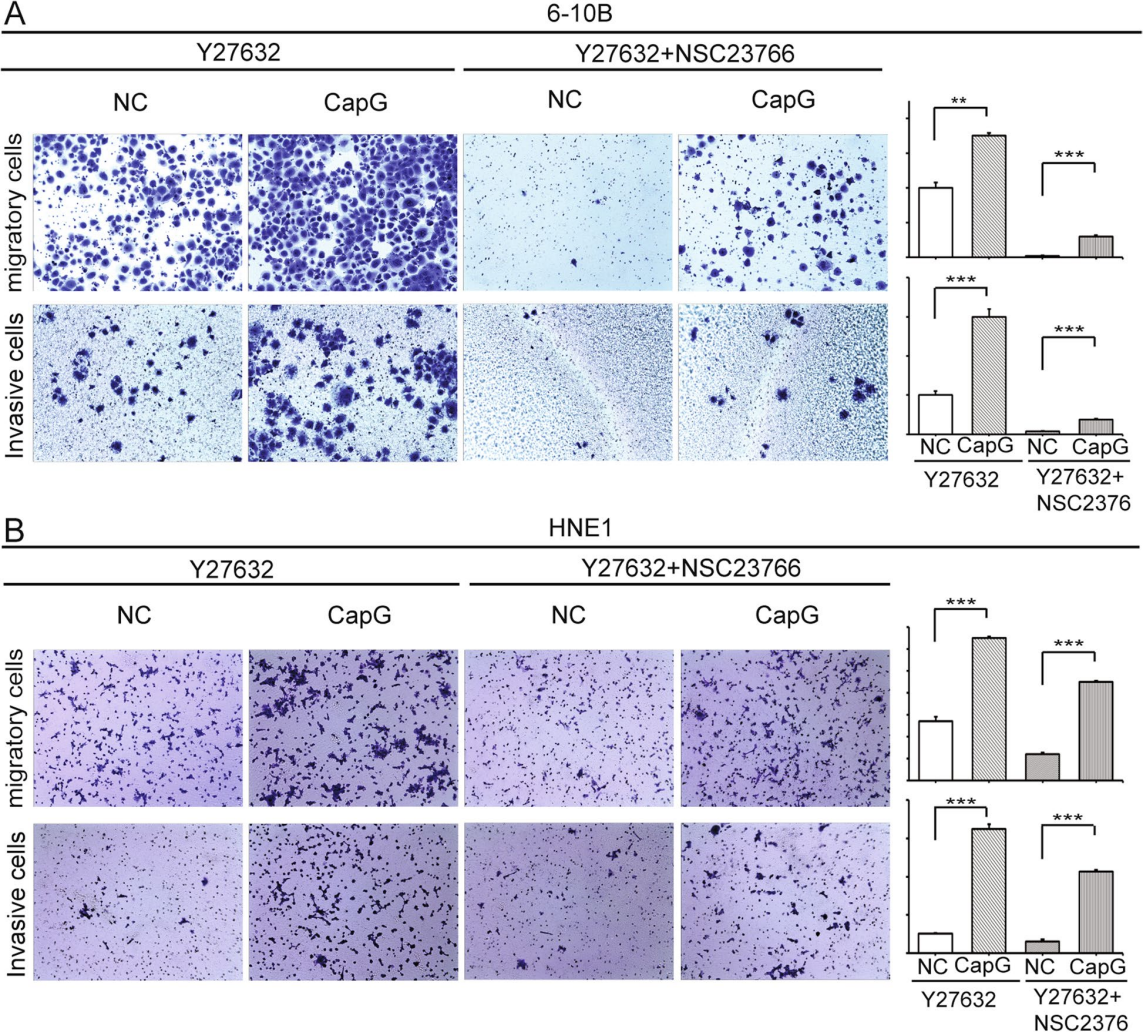
Effects of small molecular inhibitors on the motility of NPC cells. **A** Effect of Y27632 or both Y27632 and NSC23766 on migration/invasion capability of 6–10B cells. **B** Effect of Y27632 or both Y27632 and NSC23766 on migration/invasion capability of HNE1 cells. NC, negative control. **p < 0.01, ***p < 0.001

Y-27632 was used to inactivate ROCK, or both Y-27632 and NSC23766 were simultaneously adopted to inhibit both ROCK and Rac1 activity. Simultaneous inactivation of ROCK and Rac1 almost totally abolished the motility of the 6-10B NC cells in the transwell migration and invasion assay, while ectopic CapG-overexpression recovered the migration and invasion no matter in Y-27632 alone or under both the two inhibitors (Fig. 5A). Similarly, CapG-overexpressing HNE1 cells with inactivation of ROCK or with simultaneous inhibition of ROCK and Rac1 retrieved both migratory and invasive ability (Fig. 5B).

### Overexpression of CapG enhances MLC2 phosphorylation and inhibits Rac1 activation

**Fig. 6:**
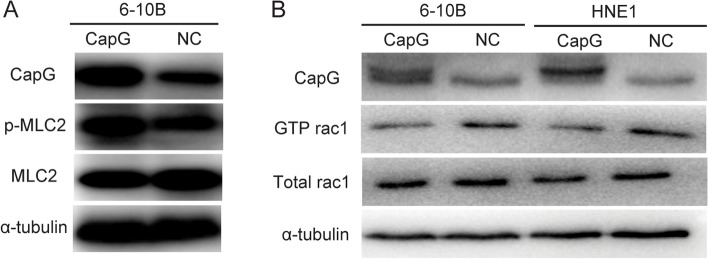
Ectopic CapG overexpression strengthened MLC2 phosphorylation and inhibited Rac1 activity. **A** Total MLC2 and phosphorylated MLC2 in CapG overexpressing 6-10B cells and the NC group. **B** Total rac1 and pulled-down GTP-rac1 in CapG overexpressing 6-10B and HNE1 cells and corresponding NC groups. α-Tubulin is used as a loading control. NC, negative control. Grouped blots were cropped from different parts of the same gel and from different exposures

MLC2 phosphorylation in CapG-overexpressing 6-10B cells was detected and found that ectopic overexpression of CapG was positively related to the Thr18 and Ser19 phosphorylation of MLC2 (Fig. 6A).

Rac1 activity (GTP Rac1/total Rac1) was detected in the ectopic CapG-overexpressing cells. As shown in Fig. 6B, when total Rac1 was not affected by ectopic CapG overexpression, GTP Rac1 was downregulated (37.49% ± 3.17%) in CapG-overexpressing 6-10B cells as compared to the NC group. Similar results of GTP Rac1 reduction (34.75.25% ± 2.25%) were observed in the CapG-overexpressing HNE1 cell line (Fig. 6B). Ectopic CapG overexpression attenuated Rac1 activity.

### CapG is positively related with Rho cell motility signaling pathway

Spearman’s correlation coefficients of CapG expression with other genes expression were calculated in the 113 fresh, treatment-naive undifferentiated NPC tumors from the GSE102349 dataset. Correlation coefficients are listed in Additional file 2: Table 1.

**Fig. 7:**
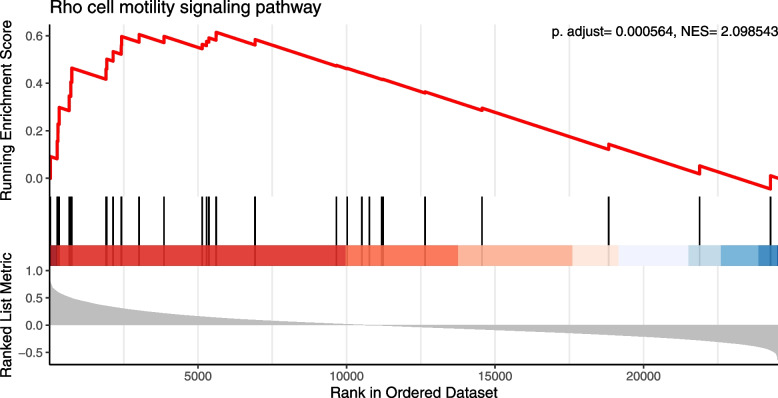
Rho cell motility pathway was positively correlated with CapG. NES, normalized enrichment score

Based on the gene list according to Spearman’s correlation coefficients, positive or negative signaling pathways associated with CapG were explored and delineated by the GSEA tool. It resulted in a total of 37 molecular pathways with statistical significance (*p* < 0.05, Additional file 3: Table 2). Most of the pathways were immune-related, whereas Rho cell motility signaling pathway is listed top among motility-related pathways (Additional file 3: Table 2). Rho cell motility signaling pathway was evidently and positively related with CapG, which contained the leading edge subset genes of RhoA and MYL2 (MLC2) (Additional file 2: Table 1, Additional file 3: Table 2, Fig. 7). In contrast, Rac1 cell motility signaling pathway did not show up in the CapG-associated significant pathways.

## Discussion

Cell motility requires spatially and temporally coordinated rearrangement of actin networks [28]. CapG is involved in shaping of cytoskeletal filaments and actin assembly through interactions with actin [28]. A meta-analysis revealed that CapG was associated with poor prognosis in a variety of malignant tumors [29]. However, it could not be ignored that CapG was identified as a tumor suppressor gene [15, 16]. Function and molecular mechanism of CapG in carcinogenesis are not completely understood.

Rho family of small GTPases, a major convergence point of migration-associated signaling, regulates the cytoskeleton and cell motility mainly in two different interconvertible movement modes depending on different usage of small GTPases signaling [30]. Rho generates contractile forces through ROCK-mediated phosphorylation of the regulatory MLC of myosin II, promoting a rounded bleb-associated mode of motility [20]. Elongated protrusive movement is associated with Rac1 activation that signals to promote the nucleation and elongation of actin filaments, which does not require Rho and ROCK [30]. Rho and Rac are mutually antagonistic as active Rac represses Rho activity and vice versa [18]. The present study confirmed that NPC motility was further more inhibited by simultaneous blockage of ROCK and Rac1 than by obstruction of ROCK alone.

Present findings indicate that CapG level was higher in the NPC cell lines or the histopathological subtypes with higher degree of malignancy which was consistent with the results of our previous quantitative and comparative proteomics study of NPC subtypes [21]. CapG existed in the stromal cells of both NNET and NPC, in accord with its prevalence in macrophages [6]. Ectopic overexpression of CapG recovered the motility of 6-10B and HNE1 cells that was obstructed by ROCK inhibitor or both ROCK and Rac1 inhibitors. Although CapG-induced invasion required RhoA [31], it was independent of ROCK, indicating that CapG exerted influence downstream of RhoA without ROCK. GSEA result confirmed that CapG was positively associated with Rho cell motility signaling pathway, rather than Rac1 cell motility signaling. In conclusion, CapG overexpression limits effectiveness of the agents that aim at reducing the motility of NPC cells through ROCK and Rac1 inhibition. Whether or not same is true, in vivo remains to be elucidated.

**Fig. 8:**
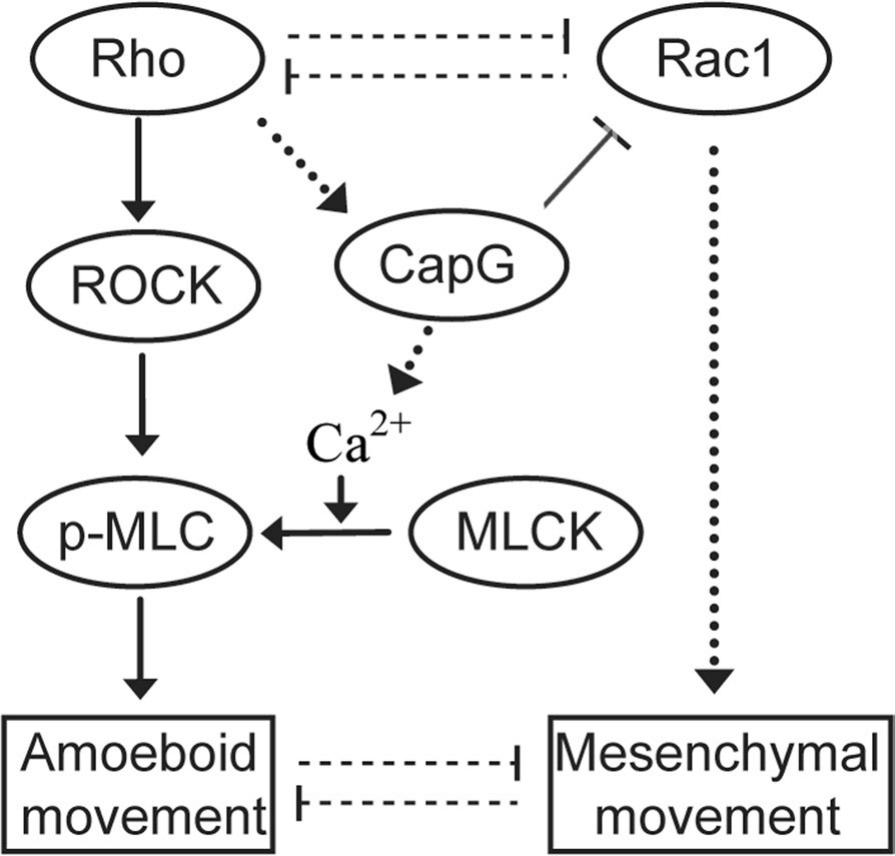
Overview of the CapG-involved motility pathway. It is derived from the present study outcomes that CapG is involved in Rho motility pathway independent of ROCK and inactivates Rac1. It is reasonably deduced that CapG-mediated Ca^2+^ signaling and Ca^2+^-dependent MLCK contribute to MLC phosphorylation. Arrows indicate activation. Truncated arrows mean inhibition. ROCK, rho-associated protein kinase; p-MLC, phosphorylated-myosin light chain; MLCK, myosin light chain kinase

In non-muscle cells, phosphorylation of myosin II has an important role in regulating actomyosin contractility [20]. Ectopic overexpression of CapG leads to more intensified phosphorylation of MLC2 in 6-10B cells. MLC is not only phosphorylated directly and indirectly by ROCK independently of Ca^2+^ but also phosphorylated by Ca^2+^-dependent myosin light chain kinase [20, 32, 33]. CapG overexpressing cell clones were found to have increased receptor-mediated phosphoinositide turnover and Ca^2+^ signaling [7]. CapG induced MLC2 phosphorylation through Ca^2+^-dependent signaling. A schematic overview is shown in Fig. 8.

Actin-myosin contractility induced by MLC2 phosphorylation led to inactivation of Rac [18] and provides a potential explanation for the suppressive effect of CapG on Rac1 activity (GTP Rac1/Total Rac1). CapG-correlated coefficient gene list also supported the hypothesis; ARHGAP22, a GTPase activator for Rac1 by converting it to inactive GDP-bound state [18], was positively and significantly correlated with CapG (*p* = 4.04e^−5^, cor. = 0.379). ARHGAP25, another Rac-specific GTPase-activating protein, was positively correlated with CapG (*p* = 1.58e^−10^, cor. = 0.553). Trio (*p* = 5.68e^−07^, cor. = −0.455) and Tiam1 (*p* = 0.06, cor. = −0.177) that catalyze exchange of GDP Rac for GTP Rac to activate the switch were negatively correlated with CapG level. RhoH, a Rac antagonist [34], was positively correlated with CapG (*p* = 1.28e^−06^, cor. = 0.442).

## Conclusion

Findings of CapG-induced MLC2 phosphorylation and Rac1 inactivation revealed role of CapG in Rho cell motility pathway. CapG promoted NPC cell motility by passing ROCK, a downstream effector of Rho, which could provide novel clues into NPC therapeutic strategies.

## Supplementary Information

**Additional file 1: Figure 1.** Efficiency of CapG knockdown or ectopic over-expression was validated by Western blotting. (A) Two pairs of double-stranded siRNA (CapG-si1 and CapG-si2) were validated for their transfection efficiency; (B) Ectopic CapG overexpressing clones were confirmed.

**Additional file 2: Table 1.** A gene list with Spearman’s correlation coefficients correlated with CapG expression level.

**Additional file 3: Table 2.** The signaling pathways that were significantly correlated with CapG level in GSEA results. GSEA: Gene Set Enrichment Analysis

## Data Availability

All data generated or analyzed during this study are included in this published article and its supplementary information files.

## Abbreviations

NPC: Nasopharyngeal carcinoma
CapG: Gelsolin-like capping actin protein
MLC2: Myosin light chain 2
ROCK: Rho-associated protein kinase
GSEA: Gene set enrichment analysis
NNET: Normal nasopharyngeal epithelial tissue
KSCC: Keratinizing squamous cell carcinoma
NKSCC: Non-keratinizing squamous cell carcinoma
NC: Negative control
NA: Nonavailable
GEO: Gene Expression Omnibus database
