# Supplementary material for: CapG promoted nasopharyngeal carcinoma cell motility involving Rho motility pathway independent of ROCK
Source: World J Surg Oncol. 2022 Oct 19;20:347. doi: 10.1186/s12957-022-02808-7 (PMC9580211; doi:10.1186/s12957-022-02808-7)
Supplement: Supplementary file 1 — Additional file 1: Figure 1. Efficiency of CapG knockdown or ectopic over-expression was validated by Western blotting. (A) Two pairs of double-stranded siRNA (CapG-si1 and CapG-si2) were validated for their transfection efficiency; (B) Ectopic CapG overexpressing clones were confirmed. [file 12957_2022_2808_MOESM1_ESM.pdf]

CapG promoted nasopharyngeal carcinoma cell motility involving Rho motility pathway independent of ROCK

Ying Fu[1, †], Xiuzhi Zhang[2, †], Xujun Liang[1], Yongheng Chen[1], Zhuchu Chen[1], Zhefeng Xiao[1, *]

[1] Department of Pathology, NHC Key Laboratory of Cancer Proteomics, National Clinical Research Center for Geriatric Disorders, Xiangya Hospital, Central South University, Changsha, 410008 Hunan, China

[2] Department of Pathology, Henan Medical College, Zhengzhou, 451191 Henan, China.

* Correspondence: xiaozf@csu.edu.cn

[†] Ying Fu and Xiuzhi Zhang contributed equally to this work.

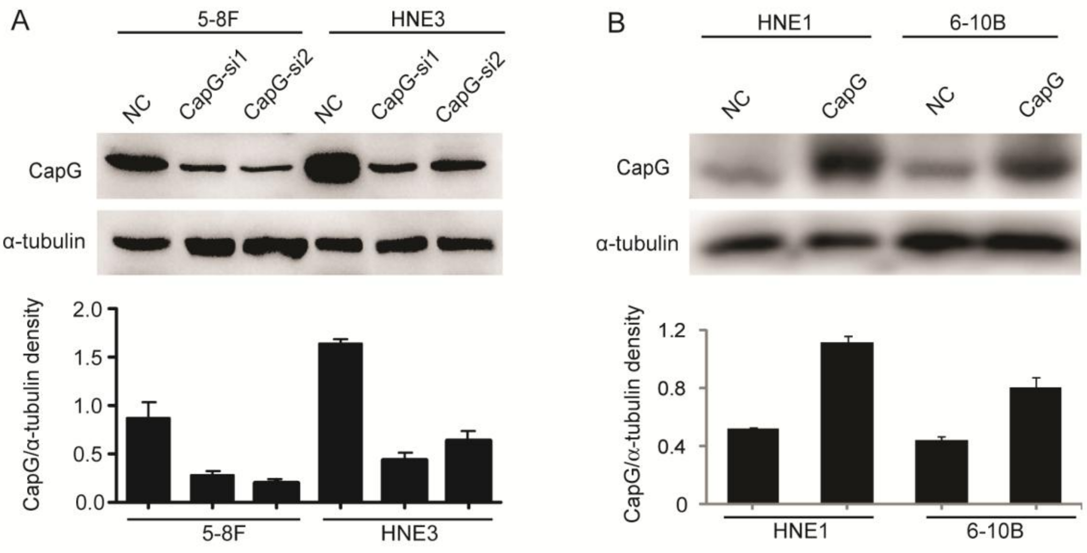

Additional file 1: Fig. 1. Efficiency of CapG knockdown or ectopic over-expression was validated by Western blotting. (A) Two pairs of double-stranded siRNA (CapG-si1 and CapG-si2) were validated for their transfection efficiency; (B) Ectopic CapG overexpressing clones were confirmed.
